# Editorial: Genomic pathways to plant health: exploring microbial symbiosis and biocontrol

**DOI:** 10.3389/fpls.2026.1898547

**Published:** 2026-06-26

**Authors:** Analía Susana Llanes, Ana A. Feregrino-Perez, Maria Doroteia Campos, Lucia Vieira Hoffmann

**Affiliations:** 1 Consejo Nacional de Investigaciones Cienitificas y Técnicas (CONICET), Instituto de Investigaciones Agrobiotecnologicas (INIAB), Universidad Nacional de Río Cuarto, Córdoba, Argentina; 2 Graduate and Research Division, Engineering Faculty, Universidad Autónoma de Querétaro, Cerro de las Campanas, Santiago de Querétaro, Querétaro, Mexico; 3 MED Mediterranean Institute for Agriculture, Environment and Development & CHANGE Global Change and Sustainability Institute, Departamento de Fitotecnia, Escola de Ciências e Tecnologia, Universidade de Évora, Évora, Portugal; 4 Brazilian Agricultural Research Corporation (Embrapa), Embrapa Algodão, Santo Antônio de Goiás, Brazil

**Keywords:** biocontrol, microbial metabolites, plant immunity, plant resilience, plant-microbe interaction, rhizosphere microbiome, signaling pathways, sustainable agriculture

Plant–microorganism interactions are central to understanding plant health, productivity, and resilience in modern agricultural systems. These interactions span a continuum from beneficial symbiosis and biocontrol to harmful pathogenic relationships. At the genomic and molecular levels, plants possess complex signaling and regulatory networks that allow them to perceive microbial cues and integrate them with defense mechanisms, redox balance, and secondary metabolism. Meanwhile, microorganisms deploy diverse strategies, including metabolite production, hormonal modulation, and activation of systemic responses, to establish interactions with plants. The integration of these processes is especially relevant in the transition toward sustainable agriculture. Beneficial plant–microbe interactions improve nutrient uptake, stress tolerance, and physiological efficiency, while reducing reliance on chemical inputs. This Research Topic aims to advance the current understanding of the genomic, transcriptomic, and molecular mechanisms that govern plant–microorganism interactions, with a particular focus on beneficial symbiosis and biocontrol. It seeks to integrate cutting-edge research on microbial communities, signaling pathways, and regulatory networks that enhance plant health, resilience, and productivity. Special attention will be given to model organisms such as *Trichoderma* spp., as well as to innovative approaches that bridge fundamental genomics with applied strategies for sustainable agriculture, fostering the development of environmentally friendly alternatives to conventional agrochemicals.

A key example is the study by Zhou et al., which developed a minimal synthetic microbial community (SynCom) composed of *Trichoderma harzianum* and *Bacillus* rugosus to control wheat crown rot caused by *Fusarium pseudograminearum*. This SynCom reshaped the rhizosphere microbiome, enhancing beneficial microorganisms while suppressing pathogenic fungi. It improved plant growth, biomass, chlorophyll content, and yield traits, while significantly reducing disease severity. Multi-omics approaches, including microbiome sequencing and metabolomics, revealed that microbial colonization altered both community structure and metabolic profiles, promoting compounds linked to plant growth and defense. This study highlights the potential of engineered microbial consortia as sustainable tools for enhancing plant health, crop protection, and agricultural productivity.

Sustainable agricultural systems also depend on maintaining and monitoring microbial community dynamics. Metagenomic approaches provide powerful tools to evaluate these changes. Monteiro et al. demonstrated how plant- and yeast-based formulations influence the grapevine leaf microbiome. Their findings showed that biological treatments increased fungal diversity compared to conventional fungicides, suggesting that microbiome management can contribute to healthier crops and reduced chemical dependence.

Another crucial symbiotic interaction is between Rhizobium bacteria and legumes, which enables biological nitrogen fixation. This process reduces the need for synthetic fertilizers and minimizes environmental impacts. Mahto et al. investigated chickpea genotypes to understand factors influencing nodulation and nitrogen fixation. They identified key physiological and molecular differences associated with efficient symbiosis, including the upregulation of the *CaNFP* gene. Their findings suggest that genome editing targeting such genes could enhance nitrogen fixation efficiency, improve plant resilience, and support sustainable farming systems.

Biocontrol strategies are also being expanded through synergistic approaches combining microorganisms and plant-derived compounds. Philip et al. studied the combined effects of *Trichoderma hamatum* and *Rumex dentatus* extracts against *Alternaria alternata* in tomato. The combination significantly reduced disease incidence and severity, outperforming conventional fungicides. It also enhanced plant growth, chlorophyll content, and antioxidant enzyme activity. These effects were linked to bioactive metabolites such as phenolics and antifungal compounds, demonstrating the potential of integrated biological strategies for to enhance plant health while effectively managing plant diseases.

Beyond fungal and bacterial pathogens, plant viruses remain a major challenge in agriculture. Elsharkawy and Alzahrani reported that *Micrococcus luteus* can induce systemic resistance against Bean yellow mosaic virus. This bacterium reduced disease severity and viral load while enhancing plant defense mechanisms, including antioxidant enzyme activity and gene expression related to defense pathways. These findings provide evidence of microbial-mediated antiviral resistance and highlight the need for further multi-omics studies to fully understand plant–microbe–virus interactions.

Arbuscular mycorrhizal fungi (AMF) represent another critical group of beneficial microorganisms. They establish symbiotic relationships with plants that facilitate nutrient exchange, strengthen plant health, and enhance tolerance to environmental stresses. Chang and Lin demonstrated that AMF-mediated drought tolerance in foxtail millet is genotype-dependent. AMF improved recovery after drought in tolerant genotypes by enhancing nutrient transport, cell wall biosynthesis, and stress signaling pathways. However, drought-sensitive genotypes showed limited benefits, emphasizing the importance of plant genetic background in determining symbiotic outcomes.

At the molecular level, transcription factors such as PLATZ proteins are emerging as key regulators of plant–microbe interactions. Studies in *Eucalyptus grandis* indicate that specific *PLATZ* genes are modulated during AMF colonization, suggesting their involvement in regulating symbiosis and plant adaptation (Liu et al.).

Overall, the integration of genomics, transcriptomics, metabolomics, and metagenomics is transforming our understanding of plant–microorganism interactions. These approaches reveal how microbial communities influence plant physiology, defense, and metabolism, and how plants respond through coordinated molecular networks. The evidence presented across these studies demonstrates that beneficial microbes can enhance plant health through multiple mechanisms, including microbiome restructuring, metabolite production, nutrient acquisition, and activation of defense pathways.

The application of these insights in agriculture offers promising opportunities for developing sustainable crop management strategies. Engineered microbial consortia, genome editing, and microbiome monitoring can reduce dependence on chemical inputs while improving crop productivity and resilience. However, challenges remain, including understanding genotype-specific responses, ensuring consistency across environments, and scaling these approaches for field applications.

In conclusion, plant–microorganism interactions represent a dynamic and complex system with significant implications for sustainable agriculture and plant health. Advances in multi-omics technologies are providing deeper insights into the molecular and ecological mechanisms underlying these interactions. Harnessing these processes through innovative strategies will be essential for enhancing plant health while achieving resilient and environmentally sustainable agricultural systems.

